# The impact of the COVID-19 pandemic on governmental hospitals performance indicators in city of Yazd, Iran: an interrupted time-series analysis

**DOI:** 10.1186/s12913-025-12587-y

**Published:** 2025-03-26

**Authors:** Mohammad Ranjbar, Mohammad Bazyar, Ommolbanin Sarkari, Hossein Ameri, Blake Angell, Yibeltal Assefa

**Affiliations:** 1 National Center for Health Insurance Research, Tehran, Iran; 2 Health Policy & Management Research Center, Department of Health Management and Economics, School of Public Health, Shahid Sadoughi University of Medical Sciences, Yazd, Iran; 3 Department of Health Management and Economics, Faculty of Health, Ilam University of Medical Sciences, Ilam, Iran; 4 Department of Health Management and Economics, International Campus of Shahid Sadoughi University of Medical Sciences, Yazd, Iran; 5 Centre for Health Systems Science, the George Institute for Global Health, University of New South Wales, Sydney, Australia; 6 School of Public Health, The University of Queensland, Brisbane, Australia

**Keywords:** Covid-19, Performance indicators, Public hospital, Interrupted time series analysis

## Abstract

**Background:**

The Covid-19 pandemic stretched health systems globally including in Iran. Hospital demand and performance was affected both directly and indirectly as a result of the pandemic. Analyzing hospital indicators can provide insights to deal with the consequences and challenges related to various aspects of future pandemics.

**Objective:**

This study aimed to investigate the impact of the Covid-19 pandemic on key performance indicators of public hospitals in Iran.

**Methods:**

In this quasi-experimental study, we used time-series analysis to examine eight key indicators of hospital performance: number of outpatient visits, number of elective hospitalization, average length of stay, hospital mortality rate, number of surgeries, hospitalization rate, emergency visits, bed occupancy rate, and hospitals’ revenue. Data were extracted from four public hospitals in Yazd at two time intervals, 15 months before and after the outbreak of COVID-19. Data were analysed using interrupted time series analysis models with STATA_17_.

**Results:**

Average length of stay (*p* = 0.02) and hospital mortality rate (*p* < 0.01) increased significantly following the outbreak of COVID-19, while the mean of other indicators such as number of outpatient visits (*p* < 0.01), number of elective hospitalization (*p* < 0.01), number of surgeries (*p* = 0.01), hospitalization rate (*p* < 0.01), emergency visits (*p* < 0.01) and bed occupancy rate (*p* < 0.01) decreased significantly. The Covid-19 pandemic had an immediately reverse significant impact on the level changes of “outpatient visits”, “elective hospitalization”, “hospitalization rate”, “emergency visits” and “bed occupancy rate” indicators (*p* < 0.05). Although the trend of surgeries indicator was affected significantly (*p* = 0.01) after the covid-19 outbreak.

**Conclusion:**

We showed significant changes in most hospital indicators after the Covid-19 pandemic, reflecting the effect of this pandemic on the performance of hospitals. Understanding the impact of a pandemic on hospital indicators is necessary for decision-makers to effectively plan an effective pandemic response and to inform resource allocation decisions.

## Background

Since late December 2019, Covid-19 has become a catastrophic threat to public health around the globe [1, 2]. The pandemic has had a great impact on the economic, social, cultural and health status of different communities [3], and placed hospitals, and health centers under significant strain. Health systems globally underwent fundamental changes to adapt to rapidly evolving circumstances and provide essential services to patients with Covid-19 and other patients. Hospitals worldwide experienced extreme disturbance in the provision of healthcare services and often dramatic escalations in the need for urgent treatment for a high number of Covid-19 patients. This had a profound impact on the performance of hospitals as the largest and most expensive operational units of the healthcare system [4–7]. For instance, in Iran, governmental hospitals took over the main load of caring for Covid-19 patients, and part of non-Covid-19 patients were directed to private sector hospitals, military hospitals, and hospitals affiliated to the Social Security Organization. On the other hand, due to public fear of catching the corona virus and avoidance of crowded and frequently-visited places including hospitals, especially hospitals designated to provide services to Covid-19 patients, the load of outpatient visits to hospitals and also receiving unnecessary services was reduced significantly. Elective surgeries were frequently cancelled over numerous peaks of Covid-19 epidemics and significant parts of hospital capacity were allocated to providing services to Covid-19 patients. Moreover, the Iranian health system sought to directly increase the capacity of hospital beds and ICUs to provide better services to Covid-19 patients. Undoubtedly, hospital performance in different domains, including the referral load and other performance indicators, has been affected [8, 9]. Various studies in different countries have demonstrated the impact of the Covid-19 pandemic on hospitals in different nations, notably affecting the load of patient visits and those receiving different services in varying ways [10, 11]. For example, studies shows that the COVID-19 pandemic has caused an abrupt reduction in the use of in-person health care, such as inpatient visits, emergency visits, and outpatient visits, which has been accompanied by a corresponding surge in the use of tele-health services simultaneously [12, 13].

The reports by the US Center for Communicable Diseases Control (CDC) at the onset of the outbreak of the Covid-19 pandemic indicate that emergency visits had decreased by 42% compared to the same period in the previous year, with the largest decrease in the load of visits occurring the age group under 14 years as well as women [14]. Hospital admissions in Croatia during the Covid-19 pandemic decreased compared to the three-year mean before the Covid-19 pandemic (2017–2019) [15]. In the UK, elective surgeries decreased by 2.3 million cases from March 2020 to February 2022, and the frequency of patients awaiting elective surgery increased to 6 million cases [16, 17]. A study by Rashdan et al. in Jordan showed that the frequency of emergency surgeries in the summer of 2020 decreased by 28% compared to the same period in 2019, when Covid-19 restrictions were not yet applied [18]. In George Hospital in London, the number of surgeries and the average length of stay decreased after national lockdown compared to the time before the national lockdown [19].

These studies in other nations, suggest that similar changes can be expected in the domain of service provision in different inpatient and outpatient wards in Iran, including financial indicators such as hospital costs and revenue sources of hospitals, efficiency indicators such as the average length of stay, bed occupancy rate, and even clinical indicators including hospital mortality rate of patients. These indicators provide useful information on the performance of hospitals, comparable over different periods of time [20]. Performance indicators are quantifiable parameters to measure performance, can help highlight factors contributing to hospital successes or shortcomings and focus attention on issues vital to the operation of hospitals now and into the future [5, 12, 13, 21].

Examining changes in hospital performance indicators following major epidemics such as Covid-19 can prepare managers to effectively manage the performance of hospitals and provide uninterrupted services to patients, as well as ensure preparedness during the ongoing threat of Covid-19 and similar pandemics in the future. Few studies have been conducted in Iran regarding the effect of the Covid-19 pandemic on the performance indicators of hospitals. Thus, considering the importance of this issue in the planning and making policies for the health system, this research investigated the impact of the Covid-19 pandemic on hospital indicators in public hospitals in Iran. The results of this research and similar studies can help managers and health policy makers to cope with emerging and re-emerging epidemics in the future.

## Methods

In this quasi-experimental study, interrupted time series analysis was applied to investigate the effects of the COVID-19 pandemic on the key indicators of public hospitals in the city of Yazd. There are ten hospitals in Yazd city including four public and four privates, one run by Social Security Organization which is one of the main social basic health insurance schemes, and the last one is affiliated with Islamic Azad University. Among public hospitals, three of which are general (named Shahid Sadoughi, Rahnamoun, and Afshar) and one is a specialized hospital. These general hospitals are big and broadly similar to each other.

### Inclusion and exclusion criteria

We included public hospitals for this study as the Ministry of Health and Medical Education and Medical Universities mainly used public hospitals to treat Covid-19 patients. Other kinds of hospitals run by non-governmental organizations such as private hospitals were excluded as they did not engage with corona patients and they do not usually provide data for research studies. We selected hospital performance indicators including number of outpatient visits and elective hospitalization, average length of stay, number of hospital mortality, number of surgeries, number of hospitalization, number of emergency visits, bed occupancy rate, and hospitals’ revenues. We chose these indicators as they are collected routinely by the medical statistics units in the hospitals as the main indicators to measure performance in hospitals and we expected to see rapid changes in these indicators as a result of COVID-19 pandemic. Furthermore, they are collected routinely in Iran and have been used by previous studies in Iran [13, 22–26]. The formula for measuring the selected indicators have been mentioned in other studies [27].

### Data collection and duration of the study

In order to access the data, a letter to introduce the subject and the research team was presented by the Research Deputy of Shahid Sadoughi University of Medical Sciences in Yazd to the governmental hospitals separately. After the letter was approved by the hospital manager and security department, the principal investigator was introduced to the “Statistics Unit” to access the data. The data for the selected indicators was received in Excel format, and on a monthly basis for a period of 30 month including a period of 15 months before (December, 2019 to February 2020) and a period of 15 months after (March 2020 to April, 2021) the COVID-19 outbreak. Then the data was transformed into the required format for analysis in a time series model. The process of data collection was done in 2022.

### Statistical methods

We used the paired t-test to investigate differences in the annual average of each indicator for the year before and after the COVID-19 pandemic. Interrupted Time Series Analysis (ITSA) is a valuable tool for evaluating interventions that occur at a specific point in time and impact an entire population [25]. It has been employed to assess a wide range of public health interventions and events, such as the effects of health programs or reforms. Additionally, ITSA has been utilized to study the impacts of unexpected events like the COVID-19 pandemic on population health trends. The versatility of ITSA allows for its broad application in retrospectively determining the effects of planned interventions or unforeseen events when randomized controlled trials are not feasible. This characteristic makes ITSA a valuable quasi-experimental methodology for evaluating interventions implemented at a population level [28, 29]. Then, we used the interrupted time series analysis method (ITS), to investigate the impact of COVID-19 pandemic on the level (immediate change) and the trend (long term change) of the indicators. In ITS, two variables indicate the amount of change due to implementing a policy or occurring an event; the first one is the level of changes showing the immediate effect of the policy and the second is the trend variable that indicates the changes in the long term. The passage of time in the present study refers to the months that have passed since the COVID-19 pandemic (March 2020 to April, 2021). ITS can reveal the probable changes (immediate and short-term changes) in the level of indicators at the beginning of the project, i.e. in the 16th month and also how the trend of each indicator has changed over time (month by month). To estimate the ITS model, according to the use of time-series data, first to prevent the false regression estimation of static data, the data of the indicators were examined using the root test of the Dickey-Fuller unit. Accordingly, the null hypothesis stating that there is a single root for all indicators is rejected (*P* < 0.05) and the time series is static for all indicators.

The following segmented regression model was used to perform ITS:

$$\begin{aligned}\text{hospital indicators}\mathrm{_t}&=\beta_0+\beta_1\text{T}_\text{t}+\beta_2 \,\text{Cov19}_\text{t}\\&+\beta_3\text{T}_\text{t}\,\text{Cov19}_\text{t}+\varepsilon_t \end{aligned}$$

In this study, the dependent variables are the selected indicators for each month. T_t_ and Cov19_t_ represent the time since the beginning of the study and a dummy variable that takes the value of zero before and one after the Covid-19, respectively. T_t_ Cov19_t_ shows the interaction effect of time and the pandemic. ε_t_ is the error term of the model. Moreover, *β*_*0*_, *β*_*1*_, *β*_*2*_, and *β*_*3*_ indicate the constant term of model, trend of each indicator without considering the Covid-19, the immediate effect (change in level) of Covid-19 on the indicators, and the long-term effect (change in trend) of the pandemic on each indicator, respectively. Stata_17_ software was used for data analysis.

## Results

Performance indicators of hospitals have changed during the Covid-19 pandemic. Although some of these changes are not statistically significant, some changes are significant. Table 1 shows that the average length of stay and hospital mortality rate in hospitals significantly increased after the Covid-19 outbreak compared to before (*P* < 0.05). At the same time, the mean of outpatient visits, elective hospitalization surgeries, the hospitalization rate, the emergency visit, and the bed occupancy rate decreased significantly after the Covid-19 outbreak (*P* < 0.05). Regarding hospital revenue, according the data, the average monthly revenue in governmental hospitals increased although it was not significant.

**Table 1:** Mean and standard deviation of hospital performance indicators before and after the Covid-19 pandemic

Indicator	15 months before pandemic		15 months after pandemic		*P*-value
	Mean	SD	Mean	SD	
Number of outpatient visits	70,327	6497	45,899	10,019	0.00
Number of elective hospitalization	4072	629	2846	624	0.00
Average length of stay	2.96	0.17	3.17	0.31	0.02
Number of hospital mortality	25	5	36	11	0.00
Number of surgeries	1448	62	878	37	0.01
Number of hospitalization	1463	136	1079	247	0.00
Number of emergency visits (N)	8040	337	5507	101	0.00
Bed occupancy rate	64.8	3.8	50.9	5.8	0.00
Average monthly revenue (billion Rial)	396	376	524	930	0.31

**Table 2:** Results of ITS model estimation for hospital performance indicators

Indicator	Variable	Coefficient	*p*-value
Number of outpatient visits	Level change due to intervention (β2)	−25983	0.00
	Trend change due to intervention (β3)	−694.5	0.43
Number of elective hospitalization	Level change due to intervention (β2)	−1743	0.00
	Trend change due to intervention (β3)	−23	0.68
Average length of stay	Level change due to intervention (β2)	0.21	0.20
	Trend change due to intervention (β3)	- 0.01	0.87
Number of hospital mortality	Level change due to intervention (β2)	33.14	0.14
	Trend change due to intervention (β3)	1.96	0.45
Number of surgeries	Level change due to intervention (β2)	8.97	0.99
	Trend change due to intervention (β3)	447	0.01
Number of hospitalization	Level change due to intervention (β2)	−2223.7	0.00
	Trend change due to intervention (β3)	8.21	0.91
Number of emergency visits	Level change due to intervention (β2)	−14473.8	0.00
	Trend change due to intervention (β3)	115.2	0.68
Bed occupancy rate	Level change due to intervention (β2)	−18.1	0.00
	Trend change due to intervention (β3)	0.47	0.46

**Fig. 1:**
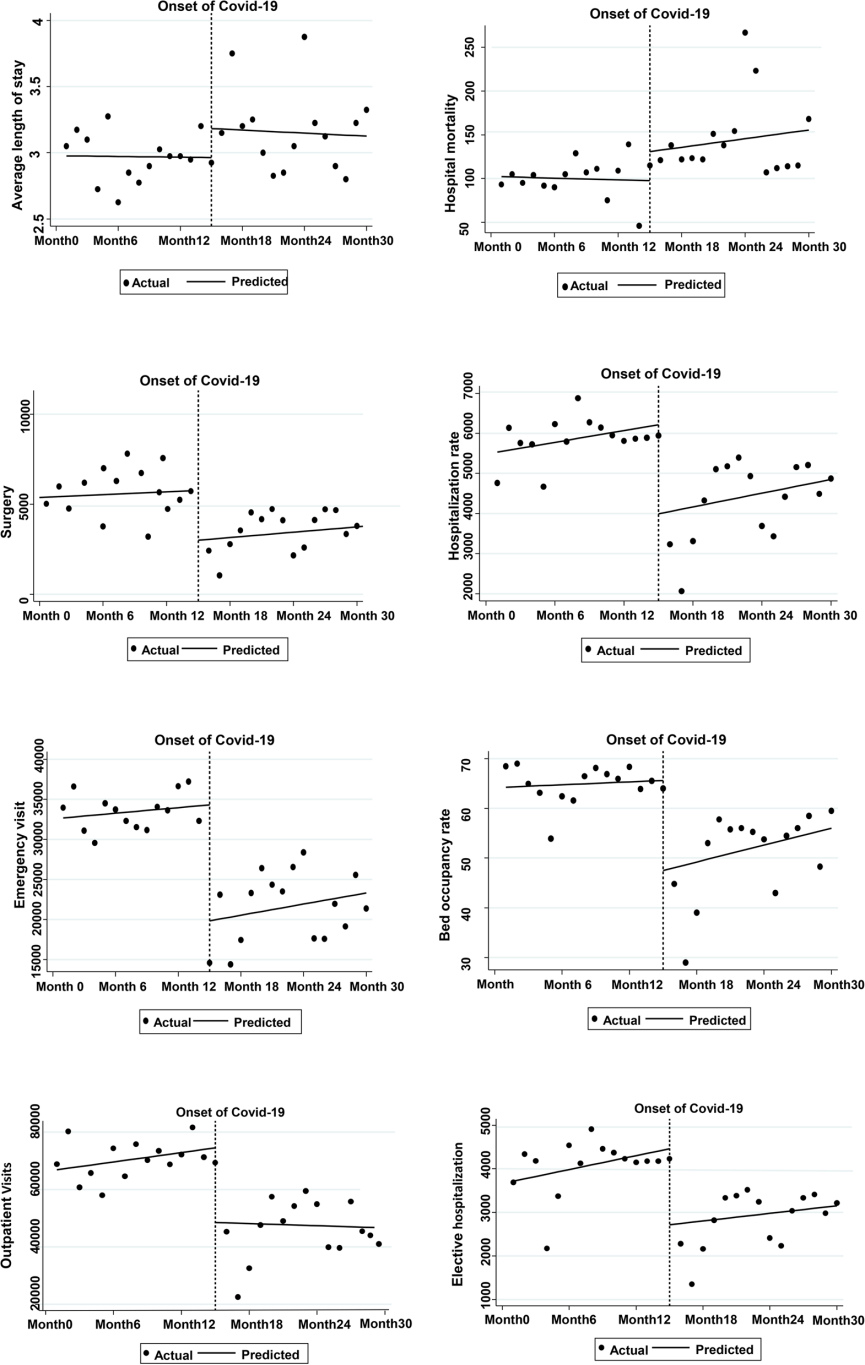
The trend of changes in selected performance indicators of hospitals in the period before and after the covid − 19 pandemic

Table 2 demonstrates that the level of indicators such as “outpatient visits”, “elective hospitalization”, “hospitalization rate”, “emergency visits”, and “bed occupancy rate” decreased significantly in the investigated hospitals after the Covid-19 pandemic, while changes in the level of other performance indicators were not significant. The trend of “outpatient visits”, “elective hospitalization”, and “average length of stay” were decreasing after the Covid-19 epidemic but not significant. Among other indicators with ascending trend, only the indicator of “number of surgeries” was statistically significant (*P* < 0.05). The highest level change after the prevalence of the Covid-19 pandemic was related to “outpatient visits (−25983)” and the lowest change was related to “average length of stay (0.21)”. The highest and lowest trend changes due to the intervention were related to “outpatient visits“(694)” and “average length of stay” (−0.01), respectively. The trends of changes in the studied indicators are shown in Fig. 1.

Regarding financial indicators such as hospital revenue, after the diligent efforts and follow-ups of the authors and obtaining administrative approvals, hospital revenues were received from the university revenue department. However, due to the delay in registering monthly revenues, this indicator was set aside from the analysis. The reason was that a portion of the revenue each month is registered in the following months for various reasons, and this was significantly higher in the last month of the year than in previous months, making it impossible to assess the impact of COVID on the monthly revenue indicator trend.

## Discussion

This study investigated the impact of the Covid-19 pandemic on the performance indicators of public hospitals in Yazd, Iran. We showed most hospital performance indicators were impacted by the Covid-19 outbreak.

Our study showed that the covid-19 epidemic led to an increase in the average length of stay at hospitals, such that immediately change after covid-19 was upward but the long-term trend was diminishing over time. Previous studies in Vietnam and India have also reported an increase in average length of stay with a descending trend during the Covid-19 pandemic [30, 31]. The results of the study by Boyle et al. in New Zealand showed that the average length of stay during the national Covid-19 quarantine period increased compared to the average length of before the implementation of quarantine [32]. Moreover, the results of Ramin Sami et al.‘s study reported an increase in the average length of stay in Isfahan hospitals in Iran during the Covid-19 pandemic [33]. Of course, some previous studies have reported a decrease in the average length of stay during the Covid-19 pandemic [19, 34–39] and others found no significant difference in the average length of stay during the Covid-19 pandemic [18]. Longer length of stay at the onset of the pandemic can be attributed to the unknown nature of the disease and the lack of treatment guidelines, and as a result, the need for longer hospitalization [40]. One reason for longer length of stay in present study can be attributed to the treating COVID-19 patients in governmental hospitals in Yazd. COVID-19 patients needed usually longer hospitalization to recover from the disease. Of course, regional differences in the average length of stay can be attributed to differences in strategies and protocols for the admission and discharge of patients in healthcare centers [40]. Even disparities in the environmental factors and demographic variables of the patients influence this issue. The availability of hospital resources and hospital beds is also one of the factors that influence this indicator [40].

We showed that, on average, hospital mortality increased significantly after the Covid-19 pandemic (*P* < 0.05). Also, the ITS results showed that the level of hospital mortality increased immediately after the outbreak of the pandemic and also in the long-term trend. One possible reason might be the treating COVID-19 patients in governmental hospitals in Yazd and higher rate of mortality among hospitalized COVID-19 patients. The findings of previous studies indicate an increase in hospital mortality rate during the Covid-19 epidemic [26, 27, 30, 33–41]. In addition, several studies have reported an increase in the mortality rate of people with cancer [27] and myocardial infarction [41], people with underlying diseases [42], as well as elderly people [43] during the Covid-19 pandemic. A study by Gash-Illescas et al. in southern Spain showed a 25.7% increase in mortality rate among non-Covid patients, although some other studies did not report any significant difference in hospital mortality during the Covid-19 pandemic [44]. There are several reasons for the differences in the results, including the differences in data sources, differences in the type of government interventions, and different methods of data analysis [36, 45–47]. The reasons behind the increased mortality will differ but it is likely that the workload imposed on hospitals during the Covid-19 pandemic was a key factor in many situations. It is also clear that delaying treatment can potentially increase the chance of mortality [34, 41, 48].

It was showed that the number of surgeries after the Covid-19 pandemic decreased significantly (*P* < 0.05) in line with previous studies [29, 40, 45, 48, 49]. Similarly, a study conducted in Auckland Hospital, New Zealand showed a 43.6% decrease in surgeries (*p* = 0.003) during the period of the Covid-19 epidemic compared to the period before the Covid-19 [38]. A study by Rashdan et al. in Jordan showed that the number of surgeries decreased by 28% in the summer of 2020 compared to the same period in 2019, when Covid-19 restrictions were not yet applied [18]. Similar studies in London and in Iran show similar findings [19, 49]. It seems that many factors have played role in reducing the number of surgeries, including the postponement of elective surgeries due to the fear of transmission of the corona virus in the hospital, changing the policies of hospitals, and imposing restrictions on elective surgeries along with national lockdown.

A significant decrease in both number of hospitalization and elective hospitalization occurred in public hospitals after the outbreak of the Covid-19 pandemic (*P* < 0.05). Further, the output of the ITS shows a significant decrease in the level of this indicator immediately after Covid-19 outbreak. The study by Patel R. et al. in southwest London showed that fewer patients were hospitalized after the quarantine of Covid-19 compared to before the quarantine [19]. The results of the study by Xiong et al. showed a decrease by 28.4%, 31.1%, and 5% in hospitalization rate during the first, second and third waves of Covid-19 in Hong Kong [37]. Decreasing number of hospitalization was shown in England and the United States as well [15, 19, 50]. The decrease in the number of hospitalized patients and elective hospitalization during the Covid-19 pandemic can be attributed to the reluctance of people to receive hospital care due to the fear of developing the Covid-19 disease, considering that the transmission of the disease is more prevalent among patients and health workers. Of course, previous studies have also generally identified the reasons for reduced load of patient visits to hospitals during the Covid-19 pandemic as not going to the hospital and postponing unnecessary care and elective surgeries due to the fear [45, 51, 52] and concern of infection with Covid-19, doctors’ reconsideration of the hospitalization of patients due to limited resources, and allocating hospital beds to Covid-19 patients [37].

Like the number of hospitalization, the number of emergency visits and outpatient visits were diminishing in public hospitals during the Covid-19 pandemic (*P* < 0.05). Also, the output of the ITS showed a significant decrease in the level of these indicators in a period of 15 months after the outbreak of the Covid-19 pandemic. The results of a study showed that there was a crowding of visits to the emergency ward and clinics of hospitals before the outbreak of the Covid-19 pandemic; yet, during the epidemic, these patterns changed in such a way that, for example, the frequency of cardiovascular patients visiting the emergency ward and also the hospitalization of these patients decreased drastically [37]. Decreased emergency admissions have been reported in many countries of the world during the Covid-19 pandemic including America and Europe [2, 52, 53]. Fear of exposure to Covid-19 in hospitals appears to be a major factor in the decline in outpatient and emergency room visits, causing many people to delay seeking care. On the other hand, the implementation of social distancing and quarantine policies has led to a reduction in road accidents, which has reduced patients’ visits to hospital emergency wards.

We also showed that a significant decrease in the bed occupancy rate after the outbreak of the Covid-19 pandemic (*P* < 0.05) so that immediately after the outbreak of Covid-19, the bed occupancy rate decreased and then increased during the period of 15-month after pandemic. Jalali et al.‘s study in Tehran revealed that the bed occupancy rate was lower one year after the pandemic compared to previous years [40]. In their study in the UK, focusing on non-Covid-19 diseases, Reschen et al. showed that hospital bed occupancy rate decreased at the beginning of the outbreak of Covid-19, but it returned to normal within a year [54]. other studies in Massachusetts [55], Tehran [40], America [50], Croatia [15] and Germany [56] showed that the hospitalization rate decreased after the Covid-19 pandemic, leading to decreased bed occupancy. It seems that the reduction of outpatient visits and, as a result, the reduction of referrals from outpatient clinics to inpatient wards, also, reduced number of non-Covid-19 patients, and the shift of focus from selective specialized care to care for emergency patients of Covid-19 are among the most important possible factors for the reduction of inpatient admissions, leading to the reduction of the bed occupancy rate in the investigated hospitals.

Regarding hospital revenues, we could not analyze the impact of the COVID-19 pandemic using ITSA, as there was a high variation in recording monthly hospital revenues. Although the average monthly revenues was higher during the COVID-19 pandemic, we could not review the immediate and short term changes in hospital revenues right after and during the first several months during COVID-19. But similar studies from Iran and other countries revealed that hospital revenue declined after outbreak of the COVID-19 pandemic at least during the first wave [26, 57, 58]. We should bear in mind that apart from health care utilization, other factors could influence the hospital revenues in Iran which coincidentally occurred almost at the same time as the onset of coronavirus. These factors include long national holidays of Nowrooz and increasing medical tariffs which have a reducing and increasing influence on revenues respectively. Another factor to consider is the average cost of each COVID-19 patient hospitalized in hospital.

### Limitations of the study

Our study had limitations. First, this study was conducted in a period of 15 months during the outbreak of the Covid-19 pandemic; thus, it highlights the changes in the outcome indicators in a short period of time. Conducting a study over a longer period of time and examining changes in indicators, especially during the peaks of the spread of Covid-19, will provide health policy-makers with a better picture of the impact of a crisis on health care. Second, this was a retrospective study and all the data needed to express the changes were not collected. There may have been unknown factors other than Covid-19 that influenced the results. Third, the data on a limited number of outcome indicators were available to researchers, and a study with more indicators, especially financial or qualitative, can show a better understanding of the impact of an epidemic on the performance of hospitals.

## Conclusion

Although we could not reveal a declining effect on hospitals’ revenues, the significant reduces utilization on surgeries, outpatient and emergency visits, an also elective hospitalizations shows that they had an adverse impact on hospital revenue. This can create a challenging condition for hospitals to manage the situation brought on by the COVID-19 pandemic, or similar crisis in the future primarily due to financial constraints and decreasing revenues. This situation highlighted the critical need for sufficient funding to maintain the quality of services provided by healthcare facilities. In response to the crisis, governments should infuse additional financial resources from the general budget into hospitals to ensure their continued operation and capacity to respond effectively to the pandemic. One effective approach to address the significant decrease in healthcare utilization is to implement a robust and sustainable electronic health system along with telemedicine services. These systems can ensure uninterrupted access to essential healthcare services for individuals during similar pandemics, without exposing them to additional health risks. Furthermore, investing in digital health infrastructure can enhance the efficiency and accessibility of healthcare services beyond times of crisis. It enables patients to receive care from the comfort of their homes, reduces the burden on physical healthcare facilities, and improves overall healthcare outcomes. Additionally, telemedicine can expand access to specialized care in remote or underserved areas, bridging gaps in healthcare disparities.

## Data Availability

The data sets generated during the study are available from the corresponding author upon reasonable request.

## Abbreviations

ICU: Intensive care unit
CDC: Center for Communicable Diseases Control
ITS: Interrupted time series
